# Overexpression of macrophage migration inhibitory factor protects against pressure overload‐induced cardiac hypertrophy through regulating the miR‐29b‐3p/HBP1 axis

**DOI:** 10.14814/phy2.16022

**Published:** 2024-06-26

**Authors:** Liang Wen, Wei Chen, Cunjun Zhu, Jie Li, Juan Zhou, Minxia Zhang, Wenqiang Zhang, Qiang Xue

**Affiliations:** ^1^ Department of Cardiology, Xijing Hospital The Fourth Military Medical University Xi'an Shaanxi China; ^2^ Department of Cardiology The 986th Hospital of Air Force Xi'an Shaanxi China

**Keywords:** cardiac hypertrophy, HMG box protein 1, macrophage migration inhibitory factor, miR‐29b‐3p, transverse aortic constriction

## Abstract

Cardiac hypertrophy is an adaptive response to stressors such as high cardiac workload, which might lead to abnormal cardiac function and heart failure. Previous studies have indicated that macrophage migration inhibitory factor (MIF) might play a protective role in cardiac hypertrophy. Here, we aimed to illustrate the mechanism of MIF in protecting against pressure overload‐induced cardiac hypertrophy. Transverse aortic constriction (TAC) mouse model was established and we found that overexpression of MIF protected against pressure overload‐induced cardiac hypotrophy in TAC treated mice, as evidenced by significantly decreased the heart weight. In addition, transthoracic echocardiography showed that overexpression of MIF restored ejection fraction in TAC‐treated mice. While TAC treatment resulted in a much larger cardiomyocyte size in mice, MIF overexpression notably decreased the cardiomyocyte size. Next, we demonstrated that MIF overexpression promoted the expression of miR‐29b‐3p which further downregulated the expression of its downstream target HMG box protein 1 (HBP1). Overexpression of HBP1 reversed the effect of MIF in alleviating Ang‐II induced oxidative stress in cardiomyocytes. In conclusion, our findings suggest that MIF could attenuate pressure overload‐induced cardiac hypertrophy through regulating the miR‐29b‐3p/HBP1 axis.

## 1 INTRODUCTION

Cardiac hypertrophy is an adaptive response to stressors such as high cardiac workload, leading to increased cardiomyocyte size, and heart wall thickness (Samak et al., 2016). Prolonged cardiac hypertrophy causes aberrant cardiac function, which increases the incidence of heart failure (Cunningham et al., 2019). A number of different factors have been identified to play essential roles in the development of cardiac hypertrophy, such as vasoactive peptides, growth factors, hormones, and catecholamines (Bisping et al., 2014; Hefti et al., 1997; Zou et al., 2002). However, the precise mechanisms underlying the induction and regulation of cardiac hypertrophy have not been elucidated thoroughly.

Macrophage migration inhibitory factor (MIF) is an important cytokine playing critical roles in regulating innate immunity and in response to stress (Calandra & Roger, 2003). Accumulating evidence has shown that MIF regulates cardiac hypertrophy (Koga et al., 2013; Xu et al., 2014, 2016). In transverse aortic constriction (TAC)‐induced mouse myocardial hypertrophy, MIF expression was enhanced and MIF antagonized myocardial hypertrophy and fibrosis via maintaining a redox homeostatic phenotype (Koga et al., 2013). Another study found that MIF deficiency exacerbated abdominal aorta constriction‐induced cardiac hypertrophy via mitigating autophagy (Xu et al., 2014). In addition, MIF was reported to protect against aging‐induced cardiac remodeling and dysfunction through ameliorating loss of autophagy (Xu et al., 2016). How MIF regulates the signaling pathways involved in cardiac hypertrophy progression and the regulatory network downstream MIF are not completely understood.

In this study, we demonstrated that overexpression of MIF protected against pressure overload‐induced cardiac hypertrophy in a TAC treated mouse model. MIF overexpression promoted the expression of miR‐29b‐3p, whose upregulation in mouse cardiomyocyte HL‐1 cells ameliorated Angiotensin‐II induced oxidative stress. Overexpression of HMG box protein 1 (HBP1), the downstream target of miR‐29b‐3p, antagonized the function of MIF in reducing oxidative stress. Taken together, MIF could attenuate pressure overload‐induced cardiac hypertrophy through regulating the miR‐29b‐3p/HBP1 axis.

## 2 MATERIALS AND METHODS

### 2.1 Animal model

Animal experiments were performed with the approval of Institutional Animal Care and Use Committee of Xijing Hospital, Air Force Medical University. Mice were obtained from Beijing Vital River Laboratory Animal Technology Co., Ltd. To establish pressure overload‐induced cardiac hypertrophy model, male 9‐week‐old mice were anesthetized by i.p. injection of a cocktail of Ketamine (100 mg/kg; Sankyo Co., Ltd, Tokyo, Japan) and xylazine (10 mg/kg; WDT, Germany), intubated, and connected to a mouse ventilator (MiniVent, Harvard Apparatus). Following midline sternotomy, a double‐blunted 27‐gauge needle was tied to the aorta between the innominate and left common carotid arteries using 6–0 silk suture. The needle was then removed, and chest and skin were closed with sutures. The corresponding sham‐operated mice underwent the same procedure without ligation of the aorta.

### 2.2 rAAV9 vector construction and administration

The coding sequence of MIF was amplified and constructed into a pAAV‐CMV‐intron‐GFP‐MCS vector (GenPharma, China). The MIF sequences were validated by DNA sequencing (Invitrogen, USA). The pAAV‐MIF vector and control vector were co‐transfected with helper vector into HEK293 cells and packed into viral vector for large‐scale production. Viral titers were measured by a Taqman qPCR assay (4453320, Applied Biosystems, USA). 1 × 10^11^ rAAV9 particles in 100 μL were administrated into mice via tail vein injection.

### 2.3 Transthoracic echocardiography

Mice were anesthetized using 2% isofluorane and transthoracic echocardiography was conducted using a Vevo 770 high‐resolution imaging system (RMV‐707B, Visual Sonics, Canada). The M‐mode echocardiogram was saved and analyzed for the diastolic and systolic ventricular septum, left ventricular volume, and left ventricular posterior wall. The ejection fraction was calculated by dividing the stroke volume by the end‐diastolic volume.

### 2.4 Cell culture

Mouse cardiomyocyte cell line HL‐1 (derived from an adult female C57BL/6J mouse) was purchased from Fenghui Biotech Co. Ltd. Cryopreserved HL‐1 cells were recovered from liquid nitrogen using cell recovery supplement (MS03‐100, TheWell bioscience, USA) and cultured in Claycomb medium (51800C, Sigma‐Aldrich, USA) supplemented with 10% fetal bovine serum (10270–106, Gbico, USA), 100 units/mL penicillin, 100 μg/mL streptomycin, 0.1 mM norepinephrine, and 2 mM L‐glutamine as described (Claycomb et al., 1998).

### 2.5 Real time‐quantitative PCR

Total RNA was purified from heart tissues or cultured cardiomyocytes using a total RNA extraction kit (RM0051, AccuRef Scientific, China) and reverse‐transcribed into cDNA using HiFiscript cDNA synthesis kit (A3500, Promega, USA). Expression of lipid metabolism associated genes was analyzed by qPCR using SYBR Green Master mix (4 309 155, Thermo, USA). GAPDH was used as an internal control for all the amplifications. The primers used in the study are listed in Table 1. These primers purified with standard desalting method were manufactured by Sangon, China.

**TABLE 1:** Sequence of qPCR primers.

Primer	Sequence (5′‐3′)
Mouse MIF forward	CTTTGTACCGTCCTCCGGTC
Mouse MIF reverse	GTGCACTGCGATGTACTGTG
Mouse ANP forward	TCTGATGGATTTCAAGAACCTGCT
Mouse ANP reverse	CGTCTCTCAGAGGTGGGTTG
Mouse BNP forward	TTGGGCTGTAACGCACTGAA
Mouse BNP reverse	TTCAAAGGTGGTCCCAGAGC
Mouse β‐MHC forward	GCTCTGAGCATTCTCCTGCTG
Mouse β‐MHC reverse	CCTTTCTCGGAGCCACCTTGG
Mouse GAPDH forward	AAGCTCATTTCCTGGTATGACA
Mouse GAPDH reverse	GAGATGCTCAGTGTTGGGGG

### 2.6 Protein immunoblotting

Proteins were isolated from heart tissues or cultured cardiomyocytes using a protein lysis buffer containing protease inhibitor cocktail (AP0231, AccuRef Scientific, China). Protein samples were quantified using a BCA protein quantification kit (AP0011, AccuRef Scientific, China) and diluted in a protein loading buffer (AP0181, AccuRef Scientific, China). Equal amount of protein sample was separated by sodium dodecyl‐sulfate polyacrylamide gel electrophoresis (SDS‐PAGE) and transferred to a 0.45 μm PVDF membrane (Millipore, USA). The membranes were probed with primary antibodies against ANP (27426‐1‐AP, Proteintech, China), BNP (ab243440, Abcam, USA), β‐MHC (10799‐1‐AP, Proteintech, China), HBP1 (11746‐1‐AP, Proteintech, China), GAPDH (10494‐1‐AP, Proteintech, China), and an HRP‐conjugated goat anti‐rabbit secondary antibody (SA00001‐2, Proteintech, China). Then, the protein expression was visualized by using ECL western blotting detection kit (AP0081; AccuRef Scientific, China).

### 2.7 Enzyme linked immunosorbent assay (ELISA)

Activities of MDA (ab238537, Abcam, USA), NADH (ab178011, Abcam, USA), and SOD (CSB‐EL022397RA, CUSABIO, USA) were measured using ELISA kits according to the manufacturer's instructions.

### 2.8 Histology and hematoxylin & eosin staining

Mouse hearts were fixed with 4% paraformaldehyde for 24 h, postfixed in different concentrations of ethanol, and subsequently embedded in paraffin. 5‐μm heart tissue sections were prepared and stained with hematoxylin & eosin (AS0016M, AccuRef Scientific, China) for routine histological analysis. The mouse heart morphology and structure were examined under a Zeiss LSM800 microscope (CarlZeiss, Germany).

### 2.9 WGA staining

Mouse hearts were harvested, perfused, and fixed in 4% paraformaldehyde followed by incubation in 30% (w/v) sucrose in phosphate‐buffered saline (PBS) overnight. Then hearts were embedded in Tissue OCT and sectioned into 8‐μm sections using a Leica CM1860 cryostat. Heart sections were stained with Alexa Fluor 488‐conjugated wheat germ agglutinin (WGA) (W11261, Invitrogen, USA) to analyze the cross‐sectional area (CSA) of the cardiomyocytes. The staining results were recorded with Zeiss LSM800 microscope (CarlZeiss, Germany).

### 2.10 Proliferation analysis

Cardiomyocyte proliferation was determined by CCK‐8 assay (AC0011, AccuRef Scientific, China) according to the manufacturer's recommendation. Briefly, 100 μL of cell suspension containing 1 × 10^4^ cells were seeded into each well of the 96‐well plate. After overnight incubation, cells were treated with Angiotensin‐II (Ang‐II) reagent. At the indicated time point, 20 μL CCK‐8 reagent was added into the cell culture for 2 h, the absorbance value of each well at 450 nm wavelength was measured by a microplate reader (Biotek, USA) for plotting a growth curve.

### 2.11 Statistical analysis

The results were presented as mean ± standard deviation. The statistical analysis was performed using GraphPad Prism (V5, Prism, USA). Student unpaired *t* test and one‐way analysis of variance (ANOVA) were used and a *p* < 0.05 was considered statistically significant.

## 3 RESULTS

### 3.1 Overexpression of MIF protects against pressure overload‐induced cardiac hypertrophy in mice

**FIGURE 1:**
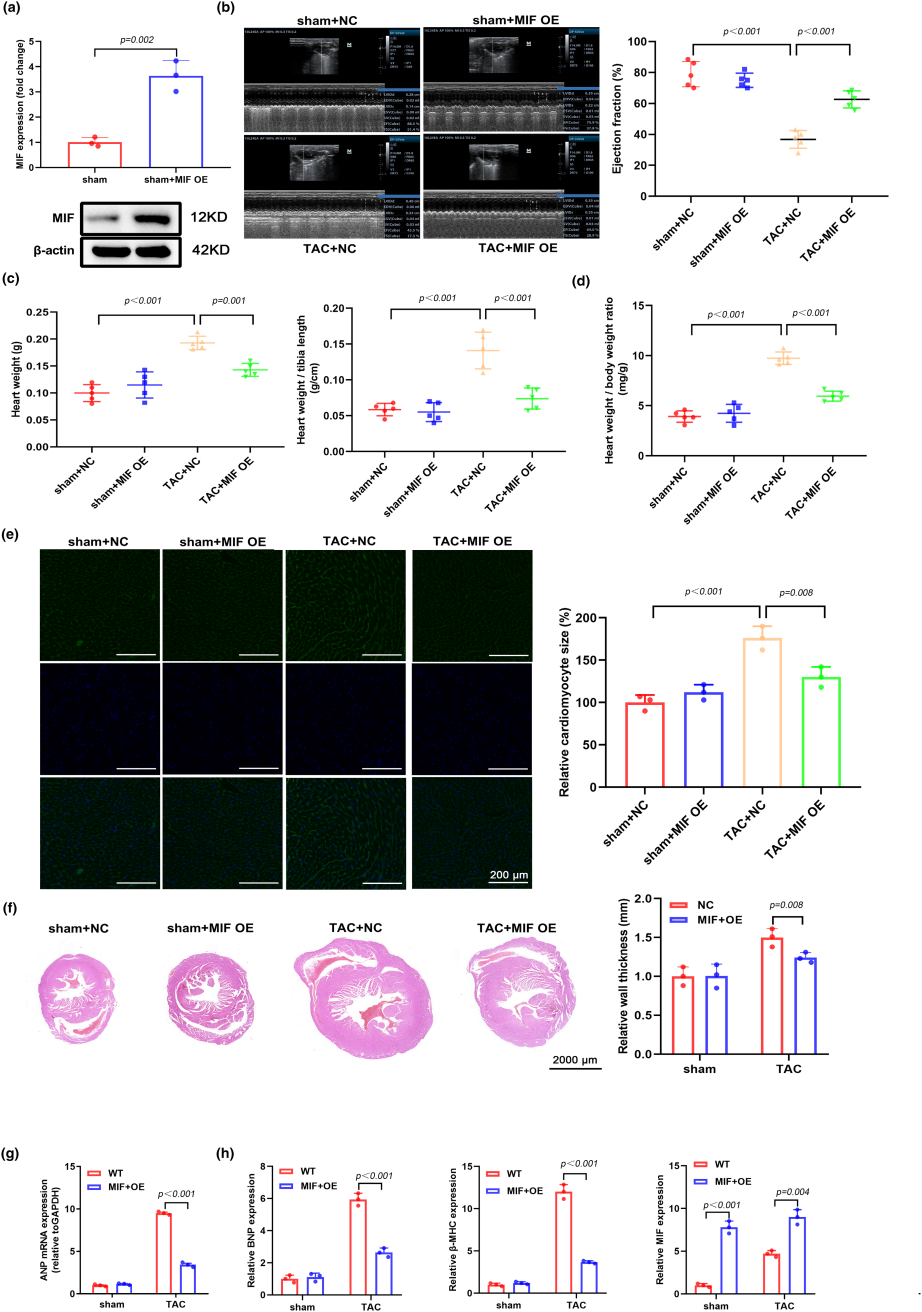
Overexpression of MIF protects against pressure overload‐induced cardiac hypertrophy in mice. Mice were received sham or TAC surgery and treated with AAV9‐overexpressing MIF (MIF OE) or control AAV9 (NC). Mice were analyzed for cardiac hypertrophy after 8 weeks. (a) MIF expression in the hearts of sham or sham+MIF OE mice was analyzed by western blot. (b) Representative images of transthoracic echocardiography are shown and the cardiac function (Ejection fraction) in different groups was analyzed. NC, negative control AAV9. The corresponding sham‐operated mice underwent the same procedure without ligation of the aorta. (c) The body weights of mice from different groups were monitored from week 7 to week 15 and the heart weights were recorded at week 15. The ratio of heart weight/tibia length was analyzed in each mouse from different groups. (d) The ratio of heart weight/body weight was analyzed in each mouse from different groups. (e) Wheat germ agglutinin (WGA) staining was performed to examine the cardiomyocyte size in different groups. (f) H&E staining was performed to show the gross morphology of mouse hearts from different groups. Scale bar, 2000 μm. The relative wall thickness was summarized. (g, h) qPCR analysis of protein expression of atrial natriuretic peptide (ANP), B‐type natriuretic peptide (BNP), β‐myosin heavy polypeptide (MHC), and MIF in different groups.

To study the function of MIF in pressure overload‐induced cardiac hypertrophy, TAC mouse model was established and cardiomyocyte‐specific overexpression of MIF was achieved via using Adeno‐associated virus serotype 9 (MIF OE). High expression of MIF was confirmed in the heart from mice transduced with MIF overexpression AAV9 vector (Figure 1a). Transthoracic echocardiography was performed at 8 weeks after surgery (Figure 1b). In comparison with the sham group, TAC treatment led to decreased ejection fraction and MIF overexpression preserved ejection fraction in TAC‐treated mice (Figure 1b). We also found that TAC‐treated mice had remarkably increased heart weight, while overexpression of MIF in TAC‐treated mice attenuated the increase of heart weight. Consistent with the changes of heart weight, TAC‐treated mice exhibited higher ratios of heart weight/body weight and heart weight/tibia length, whereas overexpression of MIF in TAC‐treated mice attenuated the increase of these ratios (Figure 1c,d). In addition, WGA staining demonstrated that mice with TAC treatment had a much larger cardiomyocyte size than mice in the sham group, while MIF overexpression notably decreased the cardiomyocyte size (Figure 1e). The cross section of the hearts showed that MIF overexpression significantly decreased the cardiomyocyte size and relative wall thickness in TAC‐treated mice (Figure 1f). We also detected the expression of hypertrophic cardiomyopathy markers such as atrial natriuretic peptide (ANP) (Figure 1g), B‐type natriuretic peptide (BNP), β‐myosin heavy polypeptide (MHC), and MIF (Figure 1h). We found that all the hypertrophic cardiomyopathy markers (ANP, BNP, and β‐MHC) were significantly increased in TAC‐treated mice and overexpression of MIF in TAC‐treated mice attenuated the increases of these markers (Figure 1g,h). Taken together, these findings suggested that overexpression of MIF ameliorated cardiac hypertrophy and its consequences in TAC‐ treated mice.

### 3.2 Overexpression of MIF attenuates Ang‐II‐induced oxidative stress in cardiomyocytes

**FIGURE 2:**
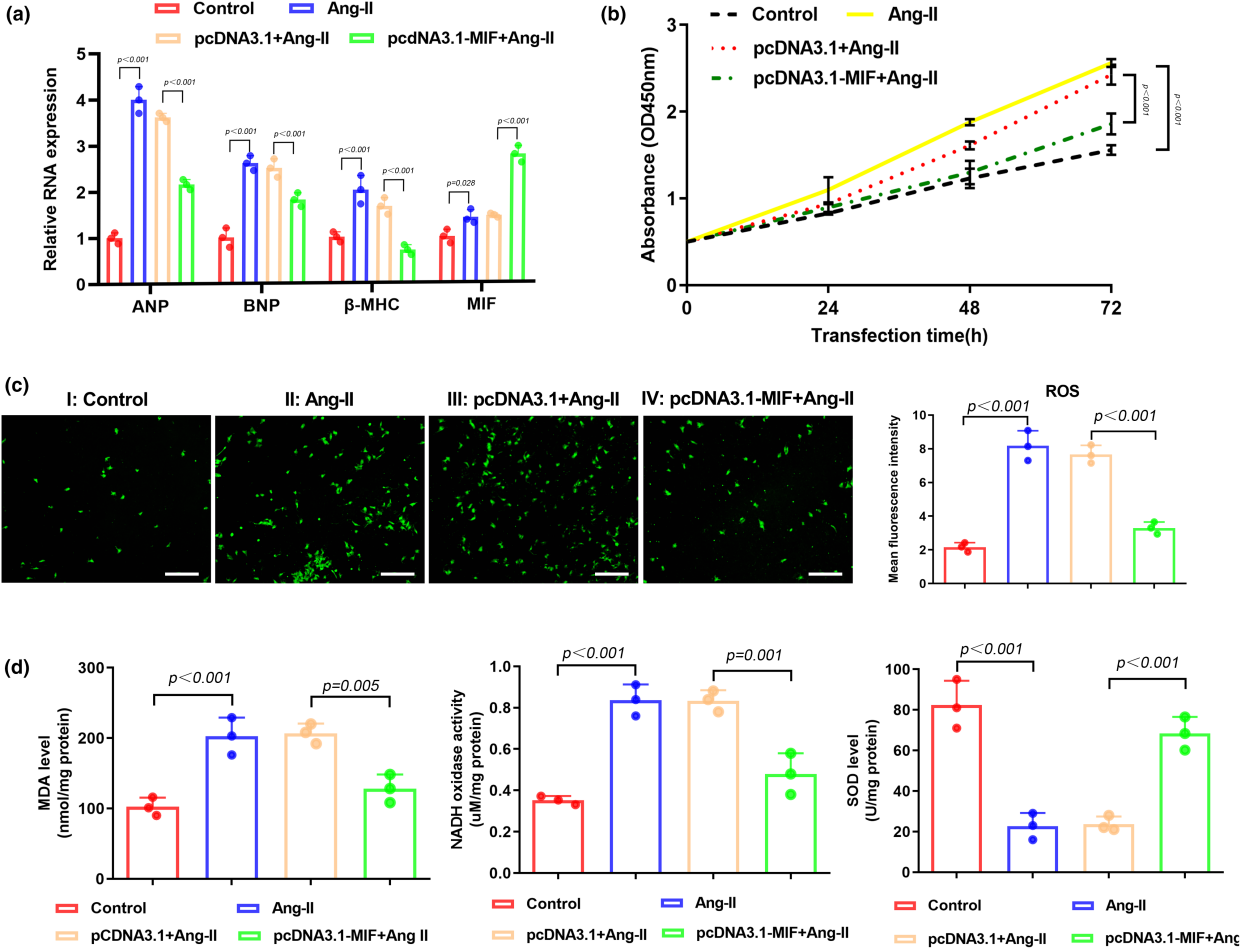
Overexpression of MIF attenuates Angiotensin‐II (Ang‐II)‐induced oxidative stress in cardiomyocytes. (a) Expressions of atrial natriuretic peptide (ANP), B‐type natriuretic peptide (BNP), β‐myosin heavy polypeptide (MHC), and MIF in HL‐1 cells after Ang II treatment were examined by qPCR. (b) CCK‐8 assay was performed at 0, 24, 48, and 72 h after transfection of HL‐1 cells with pcDNA3.1‐MIF plasmid or empty pcDNA3.1 vector. (c) DCFH‐DA staining of ROS in HL‐1 cells with different treatments. Mean fluorescence intensity was quantified using ImageJ software. Scale bar, 100 μm. (d) The activities of MDA, NADH, and SOD were detected by ELISA assay.

Previous reports have demonstrated that Ang‐II is closely associated with the occurrences of heart failure and hypertrophy (Haudek et al., 2010; Sciarretta et al., 2009). In this study we treated mouse cardiomyoblast cell line HL‐1 with Ang‐II to generate in vitro cardiac hypertrophy model. Consistent with our in vivo data, our qPCR results showed that Ang‐II‐treatment significantly increased the expression of ANP, BNP, and β‐MHC. Overexpression of MIF could remarkably decrease the levels of these markers (Figure 2a). The proliferation of HL‐1 cells showed similar trend that MIF‐treatment decreased the Ang‐II‐induced proliferation increment (Figure 2b). To evaluate the size of HL‐1 cells, we labeled α‐actin and measured cell surface area in different groups. Next, we evaluated the accumulation of intracellular reactive oxygen species (ROS) in HL‐1 cells. We found that Ang‐II‐treatment significantly increased the intracellular ROS level while overexpression of MIF could reduce the ROS level (Figure 2c). The contents of malondialdehyde (MDA) and nicotinamide adenine dinucleotide (NADH) were also reduced in the MIF‐overexpressed cells (Figure 2d). The impaired activity of superoxide dismutase (SOD) after Ang‐II‐treatment was increased in MIF‐overexpressing cells (Figure 2d). All these data suggest that MIF is a critical regulator in the removal of Ang II‐induced ROS in cardiomyocytes.

### 3.3 MIF ameliorates Ang‐II induced oxidative stress via upregulating miR‐29b‐3p

**FIGURE 3:**
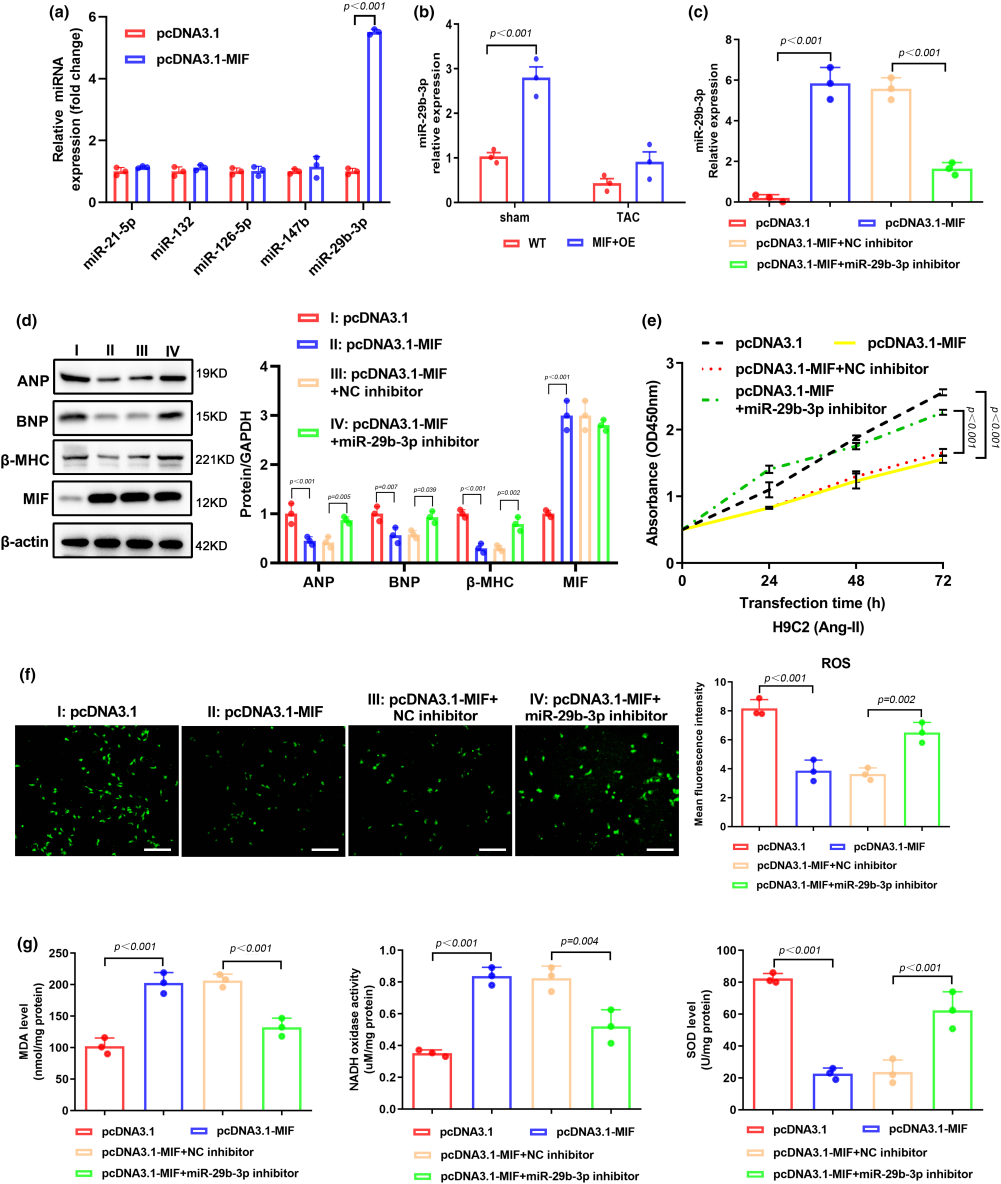
MIF ameliorates oxidative stress via upregulating miR‐29b‐3p. (a) The expression levels of pathogenic microRNAs were determined by qPCR in HL‐1 cells overexpressing MIF. (b) qPCR assay was performed to determine the level of miR‐29b‐3p in cardiac tissues. (c) The level of miR‐29b‐3p was determined by qPCR in HL‐1 cells transfected with pcDNA3.1 or pcDNA3.1 together with miR‐29b‐3p inhibitor or negative control (NC) inhibitor. (d) WB analysis of protein expression of atrial natriuretic peptide (ANP), B‐type natriuretic peptide (BNP), β‐myosin heavy polypeptide (MHC), and MIF in HL‐1 cells after the indicated transfection. (e) CCK‐8 assay measured the proliferation of HL‐1 cells after the indicated transfection of empty vector, MIF‐expressing vector, miR‐29b‐3p inhibitor, or NC inhibitor. (f) DCFH‐DA staining of ROS in HL‐1 cells with different treatments. Mean fluorescence intensity was quantified using ImageJ software. Scale bar, 100 μm. (g) The activities of MDA, NADH, and SOD were detected by ELISA assay.

Recent studies have demonstrated that miRNAs are involved in the myocardial diseases (Liang et al., 2019). We selected five miRNAs that are previously reported in the regulation of cardiac hypertrophy, namely miR‐21‐5p, miR‐29b‐3p, miR‐132, miR‐126‐5p, and miR‐147b. Our qPCR results showed that overexpression of MIF significantly increased the expressions of miR‐21‐5p and miR‐29b‐3p (Figure 3a). Since miR‐29b‐3p was the most upregulated miRNA, we further confirmed its expression in the cardiac tissues harvested from sham or TAC‐treated mice. Consistent with our in vitro data, overexpression of MIF significantly increased the level of miR‐29b‐3p in sham group (Figure 3b). To validate the regulation between MIF and miR‐29b‐3p, we treated HL‐1 cells with miR‐29b‐3p inhibitor, and found that transfection with miR‐29b‐3p inhibitor partially reversed the MIF‐induced expression of miR‐29b‐3p (Figure 3c). Functionally, our western blot results showed that ANP, BNP, and β‐MHC expressions were decreased after overexpression of MIF, and this decrement could be reversed by transfection with miR‐29b‐3p inhibitor (Figure 3d). Besides, the proliferation of MIF‐overexpressing HL‐1 cells could also be rescued by miR‐29b‐3p inhibitor (Figure 3e). As shown in Figure 3f, overexpression of MIF significantly decreased the intracellular ROS level while inhibition of miR‐29b‐3p could increase the ROS level. This finding was validated by the reduced MDA and NADH level, and increased SOD level upon miR‐29b‐3p inhibition (Figure 3g). All these data suggest that miR‐29b‐3p is regulated by MIF and plays a pivotal role in decreasing ROS accumulation.

### 3.4 HBP1 is a direct target of miR‐29b‐3p

**FIGURE 4:**
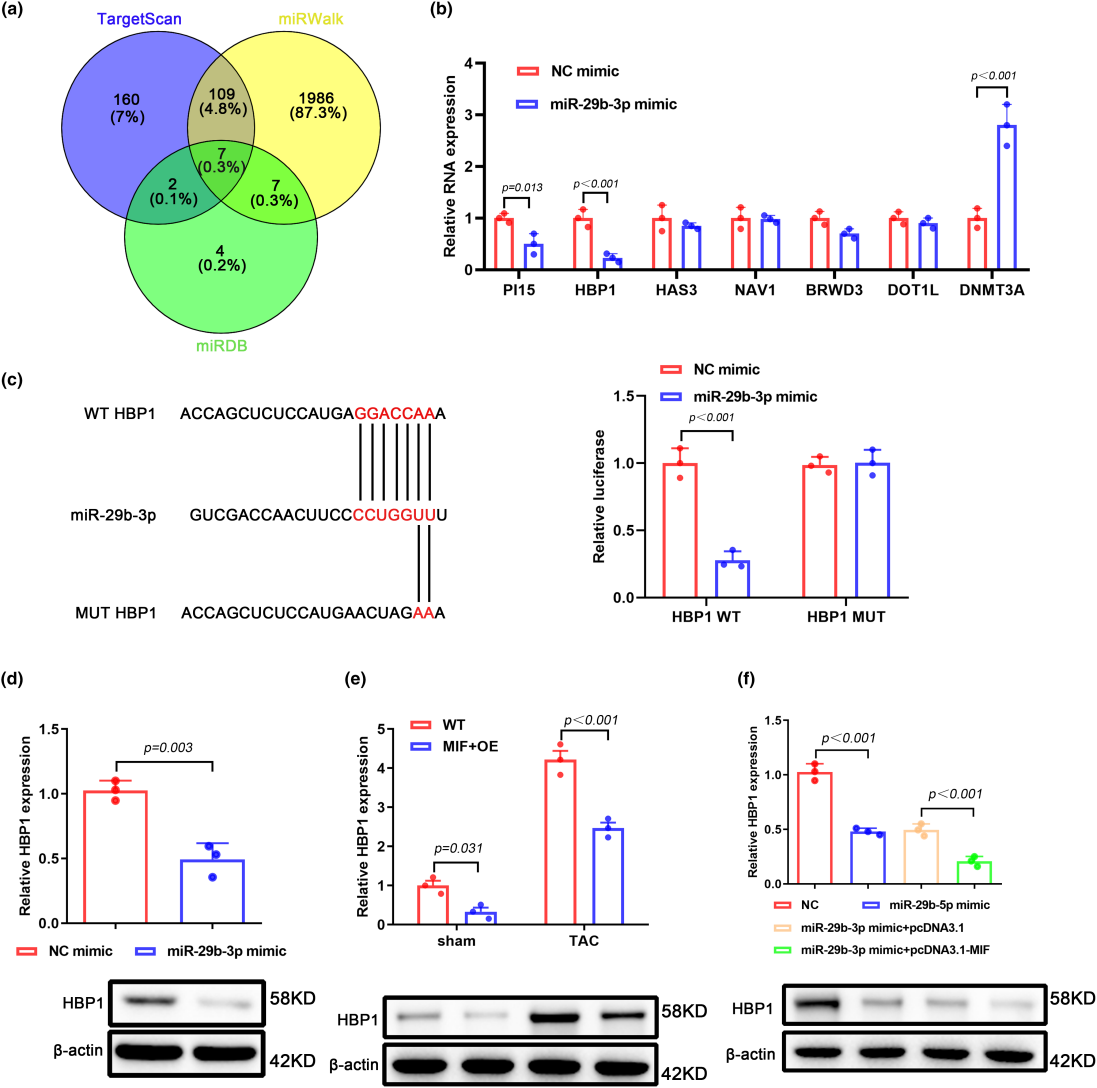
HBP1 is a direct target of miR‐29b‐3p. (a) Bioinformatic analysis of potential target genes that interact with miR‐29b‐3p. Venn diagram was generated using data acquired from TargetScan (http://www.targetscan.org), miRDB (http://mirdb.org), and miRWalk (http://mirwalk.umm.uni‐heidelberg.de). (b) qPCR detected the expression levels of targeted genes in HL‐1 cells transfected with miR‐29b‐3p mimic. (c) The binding site between HBP1 mRNA and miR‐29b‐3p. After co‐transfection of dual‐luciferase reporter plasmids and NC mimics or miR‐29b‐3p mimics into 293 T cells, dual‐luciferase reporter gene assay was used to detect the luciferase activity. (d) Expression of HBP1 in HL‐1 cells after transfection with miR‐29b‐3p mimic. (e) HBP1 expression in cardiac tissues harvested from sham group or TAC‐treated group was determined by qPCR. (f) HBP1 expression in HL‐1 cells transfected with miR‐29b‐3p mimic and/or MIF‐expressing plasmid was determined by qPCR.

We used bioinformatics tools for target prediction by multiple databases and found seven commonly predicted potential targets of miR‐29b‐3p (Figure 4a). To screen the most possible target, we transfected miR‐29b‐3p mimic into HL‐1 cells, and found that HBP1 was the most downregulated candidate gene after upregulation of miR‐29b‐3p (Figure 4b). Our dual‐luciferase assay further confirmed the interaction between miR‐29b‐3p and HBP1 (Figure 4c). Additionally, our western blot results also validated the interaction between miR‐29b‐3p and HBP1 as evidenced by significantly decreased HBP1 protein level after transfection of miR‐29b‐3p mimic (Figure 4d). Furthermore, the HBP1 level of cardiac tissues harvested from MIF‐overexpressed mice was remarkably decreased when compared with tissues from control group (Figure 4e). Similarly, co‐transfection of pcDNA3.1‐MIF plasmid and miR‐29b‐3p mimic could reduce the expression of HBP1 in a synergistical way (Figure 4f). Taken together, these data indicate that HBP1 is a direct target of miR‐29b‐3p and MIF overexpression downregulates the expression of HBP1 via upregulating miR‐29b‐3p.

### 3.5 Overexpression of HBP1 reverses the effect of MIF in alleviating Ang‐II induced oxidative stress in cardiomyocytes

**FIGURE 5:**
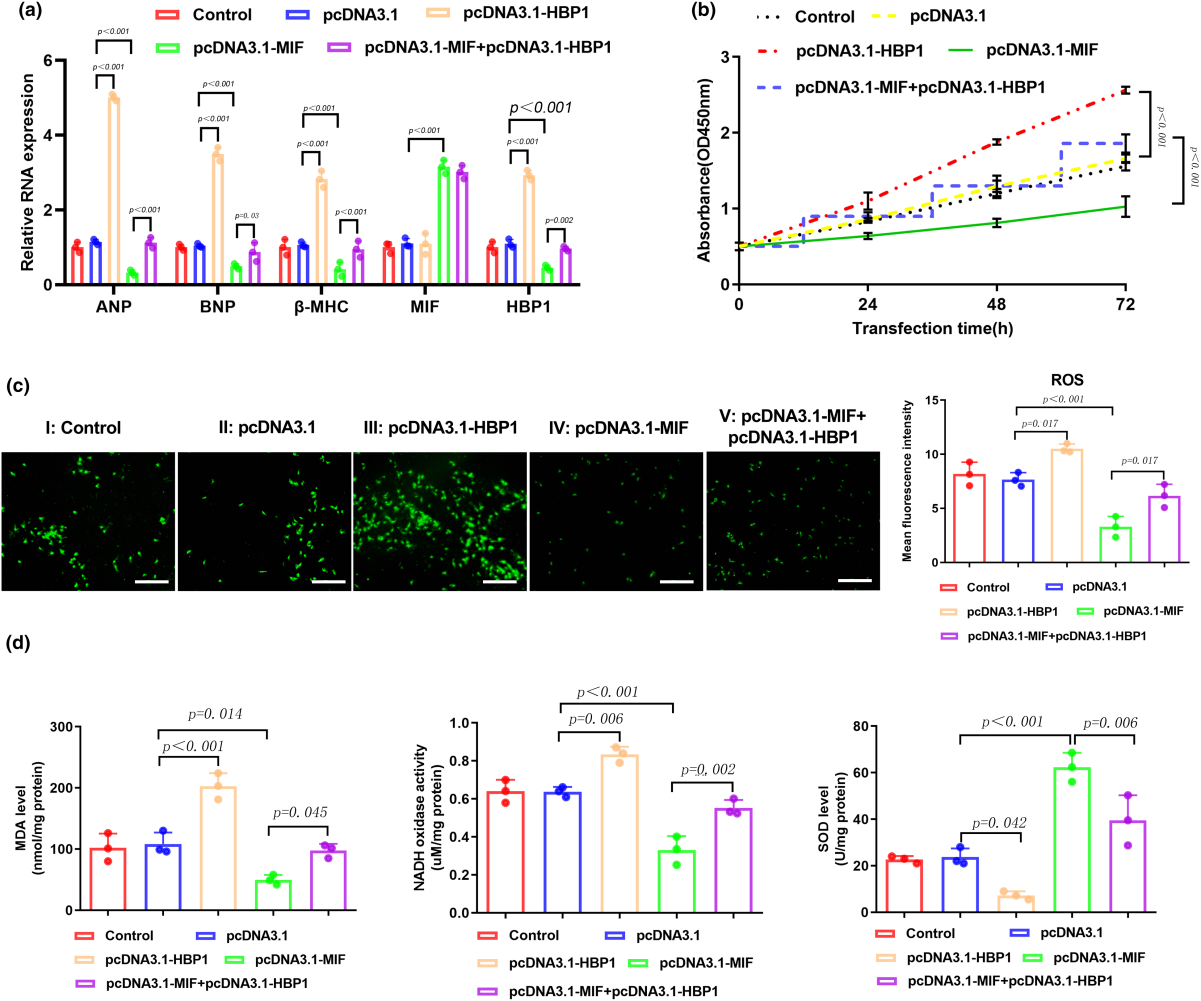
Overexpression of HBP1 reverses the effect of MIF in alleviating Ang‐II induced oxidative stress in cardiomyocytes. (a) qPCR assay was performed to determine the levels of ANP, BNP, β‐MHC, MIF, and HBP1 in HL‐1 cells after Ang II treatment. (b) CCK‐8 assay measured the proliferation of Ang II‐treated HL‐1 cells that were transfected with empty vector, HBP1‐ or MIF‐expressing plasmid, as indicated. (c) DCFH‐DA staining of ROS in HL‐1 cells with different treatments. Mean fluorescence intensity was quantified using ImageJ software. Scale bar, 100 μm. (d) The activities of MDA, NADH, and SOD were detected by ELISA assay.

To study the function of HBP1 in the pathogenesis of cardiac hypertrophy, we generated HBP1‐overexpressing HL‐1 cell line using lentivirus vector. As shown in Figure 5a, overexpression of HBP1 significantly increased the levels of cardiac hypertrophy markers ANP, BNP, and β‐MHC. Co‐transfection of pcDNA3.1‐MIF and pcDNA3.1‐HBP1 into HL‐1 cells increased the levels of ANP, BNP, and β‐MHC when compared to cells transfected with pcDNA3.1‐MIF alone (Figure 5a). Our CCK‐8 results showed that overexpression of HBP1 could increase the proliferation of HL‐1 cells with or without upregulation of MIF (Figure 5b). Next, we examined the ROS accumulation and found that overexpression of HBP1 remarkably increased the cellular ROS level (Figure 5c). Along with the upregulation of HBP1, MDA and NADH levels were increased, and the activity of SOD was decreased (Figure 5d). All these data suggest that HBP1 participates the regulation of oxidative stress in HL‐1 cells, and the abnormally upregulation of HBP1 could reverse the protective effect of MIF.

## 4 DISCUSSION

In the current study, we found that MIF played a protective role in pressure overload‐induced cardiac hypertrophy. Overexpression of MIF reduced the cardiac hypertrophy in TAC‐treated mice and reduced Ang II‐induced oxidative stress in cardiomyocytes. We also demonstrated that MIF functioned via regulating the miR‐29b‐3p/HBP1 axis. In addition, upregulation of HBP1 abrogated the protective role of MIF. These findings could provide new insights in developing therapeutics with these targets for treating cardiac hypertrophy.

MIF functions as a pro‐inflammatory cytokine but also displays antioxidant function (Farr et al., 2020). Elevated expression of MIF has been reported to be associated with all‐cause mortality in patients with heart failure (Luedike et al., 2018). Liang et al. reported that MIF inhibited ROS generation and Smad3 activation, protecting against cardiac fibrosis (Liang et al., 2019). In pressure overload induced cardiac hypertrophy, MIF protects against cardiac hypertrophy and fibrosis in response to hemodynamic stress via maintaining a redox homeostatic phenotype and attenuating stress‐induced activation of hypertrophic signaling pathways (Koga et al., 2013). Consistently, deletion of MIF exacerbated pressure overload‐induced cardiac hypertrophy via activating mTOR signaling and mitigating autophagy (Xu et al., 2014). In this study, we adopted the gain‐of‐function strategy to overexpress MIF in TAC‐treated cardiac hypertrophy mice model. We found that overexpression of MIF significantly decreased heart weight with reduced heart size in TAC‐treated mice. Therefore, combined with the published findings, MIF may play a protective role in the treatment of cardiac hypertrophy.

Altered expression of microRNAs (miRNAs) is associated with the onset of cardiac hypertrophy (Wang & Yang, 2012). Recently, miR‐29b‐3p was reported to participate in the onset of various diseases such as cancer (Lv et al., 2017), osteoarthritis (Chen et al., 2017) and cardiac fibrosis formation (Drummond et al., 2018). Roderburg et al. reported that downregulation of miR‐29b is associated with the pathogenesis of liver cirrhosis, while restoration of miR‐29b could alleviate the fibrotic score (Roderburg et al., 2011). Another study found that miR‐29b‐3p plays a protective role in cardiocytes against hypoxic‐induced apoptosis (Zhou et al., 2019). Consistent with these findings, in the present study we observed that upregulation of miR‐29b‐3p in HL‐1 cells could ameliorate Ang‐II induced oxidative stress.

In the current study, we used bioinformatics analysis to predict the potential target genes of miR‐29b‐3p and found out that HBP1 is a downstream target of miR‐29b‐3p which plays a vital role in cardiac hypertrophy. HBP1 was previously identified as a transcription factor and a tumor suppressor that inhibits the proliferation of cancer cells (Bollaert et al., 2019). He et al. reported a contradictory result that HBP1 knockdown inhibited the proliferation and metastasis of nasopharyngeal carcinoma (He et al., 2018). In our CCK‐8 assay, we found that overexpression of HBP1 significantly increased the proliferation rate of HL‐1 cells and hampered the removal of intracellular ROS. Tian et al. reported that HBP1, as a downstream target of miR‐155, regulates lipid uptake and ROS production of macrophages in atherosclerotic development by direct repression of HBP1 (Tian et al., 2014). Similarly, our results showed that the miR‐29b‐3p/HBP1 axis which is regulated by MIF plays a crucial role in the removal of intracellular ROS.

There are limitations in this study. Firstly, the interaction between MIF and miR‐29b‐3p/HBP1 axis is not fully understood. How MIF positively regulates miR‐29b‐3p expression warrants further investigation. Moreover, the regulatory mechanism of MIF in the removal of intracellular ROS is not clear. In this study we found that MIF could regulate the activity of oxidative stress‐related enzymes, but the mechanism remains unknown.

In conclusion, our findings suggest that overexpression of MIF regulates the miR‐29b‐3p/HBP1 axis and protects against pressure overload induced cardiac hypertrophy. Therefore, MIF could be utilized as a promising therapeutic target to treat the patients with cardiac hypertrophy.

## AUTHOR CONTRIBUTIONS

Liang Wen, Qiang Xue and Wenqiang Zhang conceived and designed these experiments. Wei Chen, Cunjun Zhu, Jie Li and Juan Zhou performed these experiments. Wei Chen, Cunjun Zhu, Xiamin Zhou analyzed and interpreted the data. Liang Wen, Wei Chen and Cunjun Zhu wrote the manuscript. All authors read and approved the final manuscript.

## FUNDING INFORMATION

This work was supported by National Natural Science Foundation of China (No. 81600356).

## CONFLICT OF INTEREST STATEMENT

All authors have completed the ICMJE uniform disclosure form. All the authors declare that they have no competing interests.

## ETHICS STATEMENT

Animal experiments were performed with the approval of Institutional Animal Care and Use Committee of Xijing Hospital, Air Force Medical University. We confirm that the study was reported in accordance with the ARRIVE guidelines. All methods including animals were performed in accordance with the relevant guidelines and regulations.

## Data Availability

The datasets used and/or analyzed during the present study are available from the corresponding author upon reasonable request.
